# Plasma-Derived Exosome MiR-19b Acts as a Diagnostic Marker for Pancreatic Cancer

**DOI:** 10.3389/fonc.2021.739111

**Published:** 2021-09-13

**Authors:** Lei Wang, Jinxiang Wu, Naikuan Ye, Feng Li, Hanxiang Zhan, Shihong Chen, Jianwei Xu

**Affiliations:** ^1^Department of Pancreatic Surgery, General Surgery, Qilu Hospital, Cheeloo College of Medicine, Shandong University, Jinan, China; ^2^Department of Pulmonary and Critical Care Medicine, Qilu Hospital, Cheeloo College of Medicine, Shandong University, Jinan, China; ^3^Cheeloo College of Medicine, Shandong University, Jinan, China; ^4^School of Medicine, Cheeloo College of Medicine, Shandong University, Jinan, China

**Keywords:** pancreatic cancer, liquid biopsy, exosome, miRNA, biomarker, macrophages

## Abstract

**Background:**

Diagnosis of pancreatic cancer (Pca) is challenging. This study investigated the value of plasma-derived exosome miR-19b (Exo-miR-19b) in diagnosing patients with Pca.

**Methods:**

Plasma was collected from 62 patients with Pca, 30 patients with other pancreatic tumor (OPT), 23 patients with chronic pancreatitis (CP), and 53 healthy volunteers. MiR-19b levels in plasma-derived exosomes were detected.

**Results:**

Plasma-derived Exo-miR-19b levels normalized using miR-1228 were significantly lower in Pca patients than in patients with OPT, CP patients, and healthy volunteers. The diagnostic values of Exo-miR-19b normalized using miR-1228 were superior to those of serum cancer antigen 19-9 (CA19-9) in differentiating Pca patients from healthy volunteers (area under the curve (AUC): 0.942 *vs*. 0.813, p = 0.0054), potentially better than those of CA19-9 in differentiating Pca patients from CP patients (AUC: 0.898 *vs*. 0.792, p = 0.0720), and equivalent to those of CA19-9 in differentiating Pca patients from patients with OPT (AUC: 0.810 *vs*. 0.793, p = 0.8206). When normalized using *Caenorhabditis elegans* miR-39 (cel-miR-39), Exo-miR-19b levels in Pca patients were significantly higher than those in patients with OPT, CP patients, and healthy volunteers. The diagnostic values of Exo-miR-19b normalized using cel-miR-39 were equivalent to those of CA19-9 in differentiating Pca patients from healthy volunteers (AUC: 0.781 *vs*. 0.813, p = 0.6118) and CP patients (AUC: 0.672 *vs*. 0.792, p = 0.1235), while they were inferior to those of CA19-9 in differentiating Pca patients from patients with OPT (AUC: 0.631 *vs*. 0.793, p = 0.0353).

**Conclusion:**

Plasma-derived Exo-miR-19b is a promising diagnostic marker for Pca. The diagnostic value of plasma-derived Exo-miR-19b normalized using miR-1228 is superior to that of serum CA19-9 in differentiating patients with Pca from healthy volunteers.

## Introduction

Pancreatic cancer (Pca) is a lethal disease with a 5-year survival rate of 10% and ranks as the fourth leading cause of cancer-related deaths in the United States (1). The difficulty in diagnosis of early stage diseases partly accounts for the poor prognosis of Pca. Several biomarkers for diagnosing Pca have been reported; however, most of these biomarkers have remained in the preclinical stage. Currently, only serum cancer antigen 19-9 (CA19-9) is proposed for the routine management of Pca. However, elevated CA19-9 is also observed in biliary infection or obstruction as well as other digestive cancers and inflammatory diseases and presents a moderate diagnostic value with a sensitivity and a specificity of 79% and 82%, respectively (2). Additionally, CA19-9 is not applicable for patients with negative expression of Lewis antigen, which is critical for CA19-9 biosynthesis, and the National Comprehensive Cancer Network guideline indicates that CA 19-9 is undetectable in Lewis (−) individuals (3). Notably, Luo et al. (4) reported that 8.4% of Pca patients (N = 1482) were Lewis (−). These results indicate that diagnosis of Pca based on CA19-9 will lead to missed diagnosis, and therefore, a more accurate circulating biomarker for Pca is urgently needed.

The application of liquid biopsy of circulating free DNA, tumor cells, or exosomes for cancer diagnosis has shown promise (5, 6), and among these markers, exosomes have been the subject of investigation. Exosomes are small (30–200 nm) vesicular structures that can carry pathogenic miRNAs, lncRNAs, mRNAs, DNA fragments, and proteins (7, 8). Several blood-derived exosome markers have been developed and show potential diagnostic value in Pca (5, 9). Circulating miRNAs serve as diagnostic biomarkers in multiple types of cancers, including biliary tract cancer (10), colorectal cancer (CRC) (11), Pca (5, 12), glioblastoma (13), prostate cancer (14), lung cancer (15), hepatocellular carcinoma (16), and other tumors (17). Unlike the multiple reports on circulating miRNAs, only a few studies have reported the diagnostic values of plasma- or serum-derived exosome miRNAs in Pca (18, 19).

Our previous study indicated that several plasma miRNAs were deregulated in Pca patients and presented diagnostic value^12^. Among the identified miRNAs, plasma miR-19b was significantly upregulated in patients with Pca and presented moderate diagnostic values in discriminating patients with Pca from those with chronic pancreatitis (CP) and pancreatic neuroendocrine tumor. Additionally, circulating exosomal miR-19b exhibited oncogenic functions in gastric cancer, lung adenocarcinoma, and esophageal squamous cell carcinoma (ESCC) (20–22).

In this study, we aimed to evaluate the potential diagnostic values of plasma-derived exosome miR-19b (Exo-miR-19b) in Pca. We investigated the expression levels and diagnostic values of Exo-miR-19b in patients with Pca, patients with CP, patients with other pancreatic tumor (OPT), and healthy volunteers.

## Materials and Methods

### Ethics Statement

This study was approved by the Medical Ethics Committee of Qilu Hospital of Shandong University. Written informed consent was obtained from all subjects.

### Diagnostic Criteria for Pancreatic Diseases

Pca was cytologically or pathologically diagnosed depending on the cytological or histological examinations. OPTs were pathologically diagnosed depending on histological examinations of the resected specimen, including pancreatic neuroendocrine tumor, solid pseudopapillary tumor, serous or mucinous cystadenomas, intraductal papillary mucinous neoplasms, and epithelial cysts. CP was diagnosed on the basis of clinical diagnostic criteria or histological examinations.

### Sample Collection and Exosome RNA Isolation

The serum CA19-9 levels of all included subjects could be obtained from the medical records; if not, the subjects were excluded. Pca patients undergoing neoadjuvant therapy were excluded. Peripheral venous blood (5 ml) was collected in sterile ethylene diamine tetraacetic acid-treated anticoagulant tubes before clinical intervention or surgery. The blood samples were centrifuged at 3,000 revolutions per minute (rpm) for 10 min; then plasmas were collected and stored at −80°C for further isolation of exosome. Exosomes were isolated from plasma using exoRNeasy Serum/Plasma Midi Kit (the kit can directly purify total exosomes RNA from plasma without the intermediate isolation of exosomes, Exiqon QIAGEN, #77044) according to the manufacturer’s instructions as reported by the previous study (23); then the RNA was harvested.

### Quantitative Real-Time PCR for Detecting Plasma-Derived Exo-miR-19b

Synthetic *Caenorhabditis elegans* miR-39 (cel-miR-39, RiboBio, Guangzhou, China) at 30 nM was added to each exosome RNA sample for normalization before qRT-PCR. MiRNA was converted to cDNA using a TaqMan MicroRNA Reverse Transcription Kit (Applied Biosystems). The reverse transcription reactions were carried out at 16°C for 30 min, at 42°C for 30 min, and at 85°C for 5 min and held at 4°C. cDNA was stored at −20°C until use. A total of 20 μl of amplification system containing 1.33 μl of cDNA, 10 μl of TaqMan 2× Universal PCR Master Mix with no AmpErase UNG (Applied Biosystems), 1 μl of miRNA-specific probe, and 7.67 μl of nuclease-free water was used for analyzing the expression of miRNA. qRT-PCR ran on a Stepone Plus real-time PCR system (Applied Biosystems) and the reaction mixtures were incubated at 95°C for 10 min, followed by 40 cycles at 95°C for 15 s, and 60°C for 1 min. The cycle threshold (CT) values were calculated with SDS software (Applied Biosystems). All reactions were performed in triplicate. The expression levels of miRNA were normalized using the endogenous control (miR-1228) (24) or the exogenous control (cel-miR-39). ΔCT was calculated by subtracting CT values of the miRNA from CT values of the control. The relative expression levels of miRNA were calculated with the equation 2^−ΔCT^.

### Statistical Analysis

All the statistical analyzes were performed by SPSS v.23.0 (IBM Corp., Armonk, NY). A two-sided p < 0.05 was considered as statistical significance. Continuous data were presented as the mean ± SD and analyzed using Student’s *t*-tests. Receiver operating characteristic (ROC) curves were created; and the area under the curve (AUC), sensitivity, and specificity were calculated to evaluate the diagnostic values of plasma-derived Exo-miR-19b using MedCalc Statistical Software version 19.0.4 (MedCalc Software bvba; http://www.medcalc.org). AUCs of plasma-derived Exo-miR-19b (AUC1) and CA19-9 (AUC2) were compared using Z tests.

## Results

### The Expression Levels of Plasma-Derived Exo-miR-19b in Patients With Pancreatic Cancer and Control Groups Normalized Using MiR-1228

Plasma samples were collected from 168 individuals, including 62 Pca patients, 30 patients with OPT, 23 CP patients, and 53 healthy volunteers. Exosomes RNA was extracted from the plasma samples, and Exo-miR-19b levels were determined by qRT-PCR.

**Figure 1:**
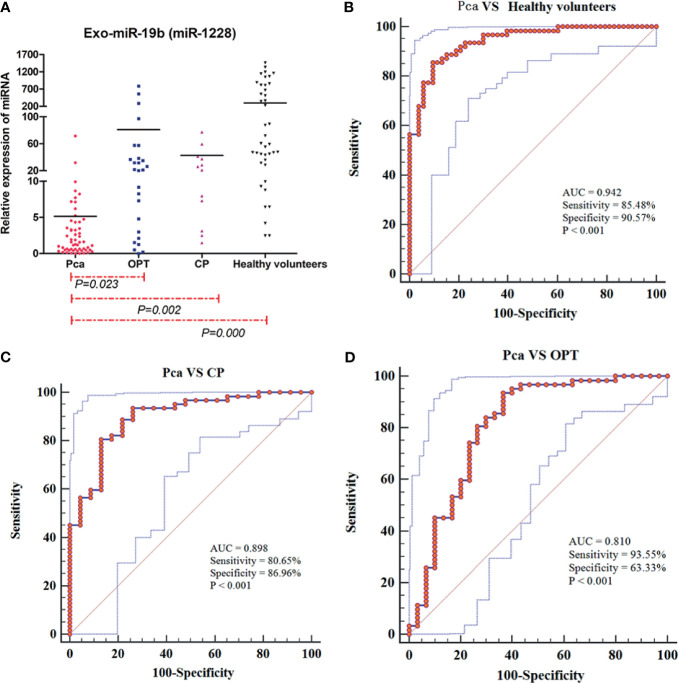
Expression levels and diagnostic values of plasma-derived Exo-miR-19b normalized using miR-1228. **(A)** Exo-miR-19b levels were detected by qRT-PCR. **(B)** ROC for differentiating Pca patients from healthy volunteers. **(C)** ROC for differentiating Pca patients from CP patients. **(D)** ROC for differentiating Pca patients from patients with OPT. AUC, area under the curve; CP, chronic pancreatitis; OPT, other pancreatic tumor; Pca, pancreatic cancer; ROC, receiver operating characteristic.

The levels of Exo-miR-19b in patients with Pca normalized using miR-1228 were significantly lower than the levels in patients with OPT, patients with CP, and healthy volunteers (p < 0.05, **Figure 1A**, **Supplementary Material 1**).

### The Diagnostic Value of Plasma-Derived Exo-miR-19b Normalized Using MiR-1228

Levels of plasma-derived Exo-miR-19b normalized using miR-1228 displayed diagnostic value in differentiating patients with Pca from patients with OPT (AUC = 0.810), patients with CP (AUC = 0.898), and healthy volunteers (AUC = 0.942) (**Figures 1B–D**, **Supplementary Material 2**).

**Figure 2:**
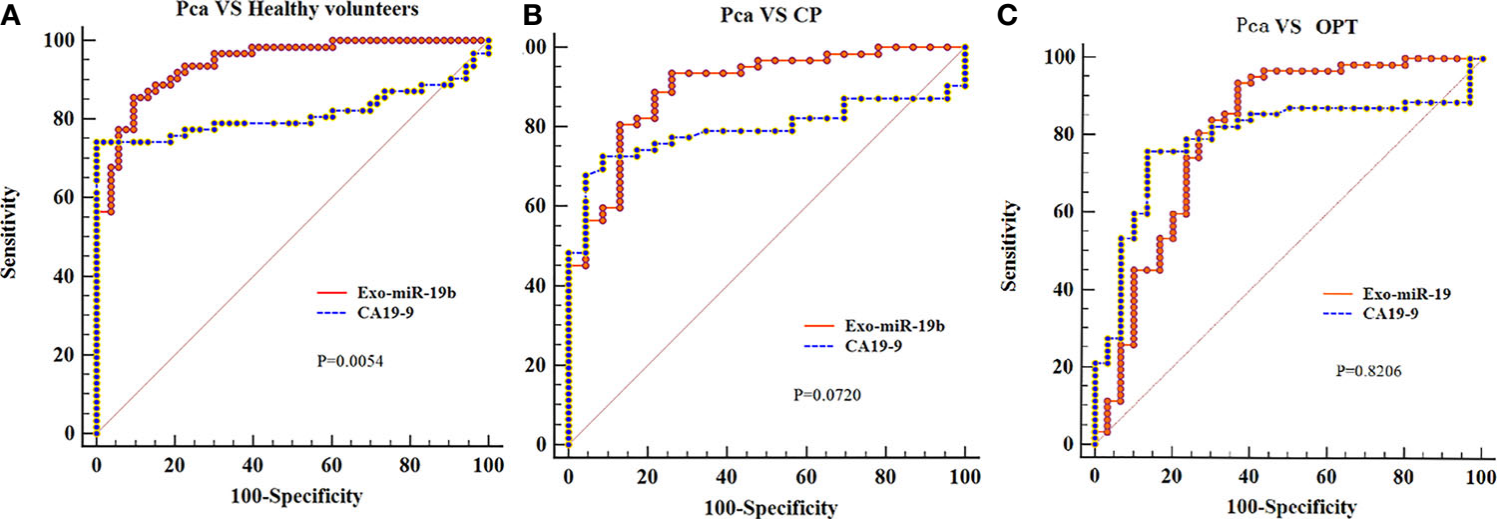
Comparison of the areas under the curves of CA19-9 with plasma-derived Exo-miR-19b levels normalized using miR-1228. **(A)** AUCs of Exo-miR-19b and CA19-9 in discriminating patients with Pca from healthy volunteers. **(B)** AUCs of Exo-miR-19b and CA19-9 in discriminating patients with Pca from CP. **(C)** AUCs of Exo-miR-19b and CA19-9 in discriminating patients with Pca from OPT. AUC, area under the curve; CP, chronic pancreatitis; OPT, other pancreatic tumor; Pca, pancreatic cancer.

Exo-miR-19b was superior to serum CA19-9 in differentiating patients with Pca from healthy volunteers (AUC: 0.942 *vs*. 0.813, p = 0.0054), potentially better than CA19-9 in differentiating patients with Pca from CP (AUC: 0.898 *vs*. 0.792, p = 0.0720), and equivalent to CA19-9 in differentiating patients with Pca from patients with OPT (AUC: 0.810 *vs*. 0.793, p = 0.8206) (**Figure 2**, **Supplementary Material 3**).

### The Diagnostic Value of Plasma-Derived Exo-miR-19b Normalized Using cel-miR-39

**Figure 3:**
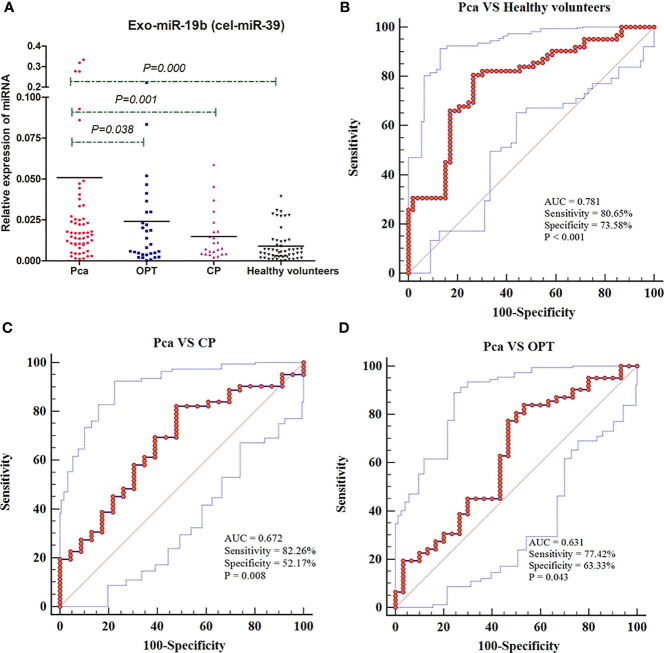
Expression levels and diagnostic values of plasma-derived Exo-miR-19b normalized using cel-miR-39. **(A)** Exo-miR-19b levels were detected by qRT-PCR. **(B)** ROC for differentiating Pca patients from healthy volunteers. **(C)** ROC for differentiating Pca patients from CP patients. **(D)** ROC for differentiating Pca patients from patients with OPT. AUC, area under the curve; CP, chronic pancreatitis; OPT, other pancreatic tumor; Pca, pancreatic cancer; ROC, Receiver operating characteristic.

The levels of Exo-miR-19b normalized using cel-miR-39 were significantly higher in patients with Pca than in patients with OPT, patients with CP, and healthy volunteers (p < 0.05, **Figure 3A**, **Supplementary Material 1**).

Levels of plasma-derived Exo-miR-19b normalized using cel-miR-39 displayed diagnostic value in differentiating patients with Pca from patients with OPT (AUC = 0.631), patients with CP (AUC = 0.672), and healthy volunteers (AUC = 0.781) (**Figures 3B–D**, **Supplementary Material 2**).

**Figure 4:**
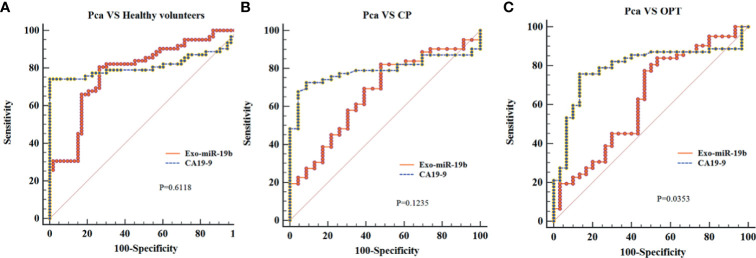
Comparison of the areas under the curves of CA19-9 with plasma-derived Exo-miR-19b levels normalized using cel-miR-39. **(A)** AUCs of Exo-miR-19b and CA19-9 in discriminating patients with Pca from healthy volunteers. **(B)** AUCs of Exo-miR-19b and CA19-9 in discriminating patients with Pca from CP. **(C)** AUCs of Exo-miR-19b and CA19-9 in discriminating patients with Pca from OPT. AUC, area under the curve; CP, chronic pancreatitis; OPT, other pancreatic tumor; Pca, pancreatic cancer.

Exo-miR-19b was equivalent to CA19-9 in differentiating patients with Pca from healthy volunteers (AUC: 0.781 vs. 0.813, p = 0.6118) and patients with CP (AUC: 0.672 *vs*. 0.792, p = 0.1235), while it was inferior to CA19-9 in differentiating patients with Pca from patients with OPT (AUC: 0.631 *vs*. 0.793, p = 0.0353) (**Figure 4**, **Supplementary Material 3**).

## Discussion

Although multiple studies are trying to find effective diagnostic markers for Pca, early diagnosis of Pca is still difficult (25). Several circulating exosomal miRNA biomarkers have been reported for the diagnosis of Pca^19^, including miR-1226 (26), miR-196a/1246 (27), miR-191/21/451a (28), and miR-10b/21/30c/161a/let-7a (29). However, few studies have investigated the diagnostic value of Exo-miR-19b (20), and no reports have been performed in Pca. In this study, we showed that plasma-derived Exo-miR-19b level normalized using miR-1228 was superior to that using serum CA19-9 in differentiating patients with Pca from healthy volunteers, potentially better than that using CA19-9 in differentiating patients with Pca from patients with CP, and equivalent to that using CA19-9 in differentiating patients with Pca from patients with OPT.

The selection of an appropriate endogenous control for normalization of circulating miRNA expression is crucial for obtaining reliable data. Several miRNAs are commonly used as endogenous controls for quantifying circulating miRNAs, such as miR-16, miR-223, let-7a, and RNU6B (24, 30). However, none of the endogenous miRNAs have been widely accepted. Our study used endogenous miR-1228 as a control for the quantification of plasma-derived Exo-miR-19b. MiR-1228 is widely involved in metabolism-related signalling pathways and organ morphology and not influenced by hemolysis (30, 31), indicating the suitability of miR-1228 as a housekeeping miRNA. Hu et al. (24) examined a large cohort of 544 subjects to identify a stable endogenous control for the quantification of circulating miRNAs in cancer patients. The authors found that miR-1228 functioned as a housekeeping gene and was stable in plasma samples from different kinds of tumors, including hepatocellular cancer, CRC, lung cancer, ESCC, gastric cancer, renal cancer, prostate cancer, and breast cancer. Duran-Sanchon et al. (31) verified the housekeeping role of miR-1228 in CRC by a large sample study; the authors showed that miR-1228 was an adequate endogenous control for circulating miRNA analysis in CRC and demonstrated a variability and stability superior to that of miR-16. Danese et al. (30) reported the expression levels and stability of miR-1228; the study found that miR-1228 displayed median PCR-derived cycle threshold values and was sufficiently homogenous and stable in exosomes, plasma, and tissues from CRC patients and healthy controls.

Previous studies on miR-19b functions indicated its oncogenic role (32, 33), suggesting that plasma-derived Exo-miR-19b would be upregulated in Pca patients compared with controls. However, our study found conflicting results. While the plasma-derived Exo-miR-19b level in Pca patients was significantly lower than that in the control subjects when normalized using miR-1228, the plasma-derived Exo-miR-19b in Pca patients was significantly higher than that in controls when normalized using cel-miR-39, which was consistent with the literature (20, 21). While the mechanism and reason underlying the differences in these results are not yet clear, some potential explanations are possible. First, some studies reported miR-1228 as a functional miRNA (34, 35), which might influence its value as an internal control. The role of miR-1228 in Pca has not been investigated, and the housekeeping role and the value of normalization for plasma-derived exosome miRNA need further experimental verification. Second, the use of an exogenous control has limitations. Exogenous controls have shown utility for quality control for RNA extraction and PCR, but useless in quality control for exosome extraction. However, quality control during extraction is important for exosome studies. Because of the differences between the expression profiles of miRNAs in plasma, serum, and blood cells (36), quality control of exosome extraction is necessary to eliminate the influence of blood components. Finally, the source of plasma-derived Exo-miR-19b is unclear. Besides Pca cells, blood cells, bone marrow mesenchymal stem cells, endothelial cells, and other cells secrete Exo-miR-19b [4-6]. The function of miR-19b or Exo-miR-19b in Pca cells might be not equal to that of plasma-derived Exo-miR-19b in Pca patients. Further studies are necessary to investigate the biological functions of plasma-derived Exo-miR-19b in Pca patients.

This study has some limitations. The control diseases are not comprehensive; acute pancreatitis, obstructive jaundice caused by benign diseases, and other digestive cancers are no included. CP is diagnosed according to either the clinical criteria or histological examinations. However, CP has a potential of malignant transformation; diagnosis with clinical criteria might miss cases with focal cancerization. The sample size is moderate; there is no stratified analysis of Pca cases; influences of jaundice, and locations and stages of the tumors on the levels of plasma-derived Exo-miR-19b are unknown. A larger sample muticenter study is helpful to disclose the diagnostic value of plasma-derived Exo-miR-19b.

In conclusion, we reported the diagnostic value of plasma-derived Exo-miR-19b in Pca. Our results showed that plasma-derived Exo-miR-19b level normalized using miR-1228 was superior to serum CA19-9 in differentiating patients with Pca from healthy volunteers, potentially better than CA19-9 in differentiating patients with Pca from patients with CP, and equivalent to CA19-9 in differentiating patients with Pca from patients with OPT.

## Data Availability Statement

The original contributions presented in the study are included in the article/**Supplementary Material**. Further inquiries can be directed to the corresponding authors.

## Ethics Statement

The studies involving human participants were reviewed and approved by the Medical Ethics Committee of Qilu Hospital of Shandong University. The patients/participants provided their written informed consent to participate in this study.

## Author Contributions

JX proposed and designed the study. LW and JW wrote the draft. LW, JW, NY, FL, HZ, and SC collected and analyzed the data. All authors contributed to the design and interpretation of the study and to further drafts. JX and FL revised the manuscript. All authors contributed to the article and approved the submitted version.

## Funding

This study was supported by grants from the National Natural Science Foundation of China (81502051), the Shandong Provincial Natural Science Foundation, China (ZR2020MH256), Medical Health Science and Technology Project of Shandong Provincial Health Commission (2019WS386), Key Technology Research and Development Program of Shandong (2019GSF108065), and the China Postdoctoral Science Foundation (2018M632681).

## Conflict of Interest

The authors declare that the research was conducted in the absence of any commercial or financial relationships that could be construed as a potential conflict of interest.

## Publisher’s Note

All claims expressed in this article are solely those of the authors and do not necessarily represent those of their affiliated organizations, or those of the publisher, the editors and the reviewers. Any product that may be evaluated in this article, or claim that may be made by its manufacturer, is not guaranteed or endorsed by the publisher.
