# Supplementary material for: Plasma-Derived Exosome MiR-19b Acts as a Diagnostic Marker for Pancreatic Cancer
Source: Front Oncol. 2021 Sep 13;11:739111. doi: 10.3389/fonc.2021.739111 (PMC8473875; doi:10.3389/fonc.2021.739111)
Supplement: Supplementary file 1 [file Table_1.docx]

**Supplementary Material 1 The expression levels of plasma-derived Exo-miR-19b**

| Group | Exo-miR-19b | P value | | |
| --- | --- | --- | --- | --- |
|  |  | **Pca VS other groups** | **OPT VS other groups** | **CP VS other groups** |
|  | **Normalisation using miR-1228** | | | |
| Pca (n=62) | 5.13 ± 10.31 | - | 0.023 | 0.002 |
| OPT (n=30) | 80.98 ± 173.56 | 0.023 | - | 0.258 |
| CP (n=23) | 42.66 ± 49.69 | 0.002 | 0.258 | - |
| Healthy volunteers (n=53) | 295.75 ± 434.80 | 0.000 | 0.002 | 0.000 |
|  | **Normalisation using cel-miR-39** | | | |
| Pca (n=62) | 0.051 ± 0.080 | - | 0.038 | 0.001 |
| OPT (n=30) | 0.024 ± 0.042 | 0.038 | - | 0.315 |
| CP (n=23) | 0.015 ± 0.015 | 0.001 | 0.315 | - |
| Healthy volunteers (n=53) | 0.009 ± 0.010 | 0.000 | 0.060 | 0.048 |
