# Supplementary material for: Plasma-Derived Exosome MiR-19b Acts as a Diagnostic Marker for Pancreatic Cancer
Source: Front Oncol. 2021 Sep 13;11:739111. doi: 10.3389/fonc.2021.739111 (PMC8473875; doi:10.3389/fonc.2021.739111)
Supplement: Supplementary file 2 [file Table_2.docx]

**Supplementary Material 2 The diagnostic values of plasma-derived Exo-miR-19b-3p**

| Group | AUC | 95% CI | Criterion | Sensitivity | Specificity | P value |
| --- | --- | --- | --- | --- | --- | --- |
|  | **Normalisation using miR-1228** | | | | | |
| Pca VS Healthy volunteers | 0.942 | 0.883-0.977 | ≤8.66 | 85.48% | 90.57% | <0.0001 |
| Pca VS CP | 0.898 | 0.813-0.953 | ≤7.18 | 80.65% | 86.96% | <0.0001 |
| Pca VS OPT | 0.810 | 0.714-0.884 | ≤12.97 | 93.55% | 63.33% | <0.0001 |
|  | **Normalisation using cel-miR-39** | | | | | |
| Pca VS Healthy volunteers | 0.781 | 0.694-0.853 | >0.0096 | 80.65% | 73.58% | <0.0001 |
| Pca VS CP | 0.672 | 0.561-0.770 | >0.0077 | 82.26% | 52.17% | 0.0080 |
| Pca VS OPT | 0.631 | 0.524-0.729 | >0.01013 | 77.42% | 53.33% | 0.0427 |
