# Supplementary material for: Plasma-Derived Exosome MiR-19b Acts as a Diagnostic Marker for Pancreatic Cancer
Source: Front Oncol. 2021 Sep 13;11:739111. doi: 10.3389/fonc.2021.739111 (PMC8473875; doi:10.3389/fonc.2021.739111)
Supplement: Supplementary file 3 [file Table_3.docx]

**Supplementary Material 3 The comparative analysis on the diagnostic values of plasma Exo-miR-19b-3p and CA19-9**

| Group | Exo-miR-19b | | CA19-9 | | z statistic | P value |
| --- | --- | --- | --- | --- | --- | --- |
|  | **AUC** | **95% CI** | **AUC** | **95% CI** |  |  |
|  | **Normalisation using miR-1228** | | | | | |
| Pca VS Healthy volunteers | 0.942 | 0.883-0.977 | 0.813 | 0.730-0.880 | 2.782 | 0.0054 |
| Pca VS CP | 0.898 | 0.813-0.953 | 0.792 | 0.691-0.873 | 1.799 | 0.0720 |
| Pca VS OPT | 0.810 | 0.714-0.884 | 0.793 | 0.696-0.871 | 0.227 | 0.8206 |
|  | **Normalisation using cel-miR-39** | | | | | |
| Pca VS Healthy volunteers | 0.781 | 0.694-0.853 | 0.813 | 0.730-0.880 | 0.508 | 0.6118 |
| Pca VS CP | 0.672 | 0.561-0.770 | 0.792 | 0.691-0.873 | 1.540 | 0.1235 |
| Pca VS OPT | 0.631 | 0.524-0.729 | 0.793 | 0.696-0.871 | 2.105 | 0.0353 |
